# A study on ship collision conflict prediction in the Taiwan Strait using the EMD-based LSSVM method

**DOI:** 10.1371/journal.pone.0250948

**Published:** 2021-05-10

**Authors:** Tian Chai, Han Xue

**Affiliations:** Navigation Institute, Jimei University, Xiamen, China; Torrens University Australia, AUSTRALIA

## Abstract

Ship collision accidents are the primary threat to traffic safety in the sea. Collision accidents can cause casualties and environmental pollution. The collision risk is a major indicator for navigators and surveillance operators to judge the collision danger between meeting ships. The number of collision accidents per unit time in a certain water area can be considered to describe the regional collision risk However, historical ship collision accidents have contingencies, small sample sizes and weak regularities; hence, ship collision conflicts can be used as a substitute for ship collision accidents in characterizing the maritime traffic safety situation and have become an important part of methods that quantitatively study the traffic safety problem and its countermeasures. In this work, an EMD-QPSO-LSSVM approach, which is a hybrid of empirical mode decomposition (EMD) and quantum-behaved particle swarm optimization (QPSO) optimized least squares support vector machine (LSSVM) model, is proposed to forecast ship collision conflicts. First, original ship collision conflict time series are decomposed into a collection of intrinsic mode functions (IMFs) and a residue with EMD. Second, both the IMF components and residue are applied to establish the corresponding LSSVM models, where the key parameters of the LSSVM are optimized by QPSO algorithm. Then, each subseries is predicted with the corresponding LSSVM. Finally, the prediction values of the original ship collision conflict datasets are calculated by the sum of the forecasting values of each subseries. The prediction results of the proposed method is compared with GM, Lasso regression method, EMD-ENN, and the predicted results indicate that the proposed method is efficient and can be used for the ship collision conflict prediction.

## 1. Introduction

The global shipping industry is witnessing a boom as economic globalization gains speed and the world economic integration trend intensifies in recent decades. According to the Review of Maritime Transport 2019, about 90 percent of global trade in terms of the weight of goods is undertaken by shipping, there is no doubt that shipping plays an irreplaceable role in the global economy [1]. However, shipping has long been regarded as a complex and high-risk activity, and maritime accidents often lead to serious damage, death, loss, injury or pollution, and may also have significant political, economic and environmental consequences [2]. The greater the role that shipping plays in international trade, the greater the impact on the world economy from the loss arising from maritime accidents. There are various international safety regulations to regulate the operation of ships and prevention of accidents, such as SOLAS 74/78/88 (International Convention for the Safety of Life at Sea), MARPOL 73/78 (Marine Pollution), STCW 78 (Standards of Training, Certification and Watch keeping for Seafarers) and COLREG 72 (International Regulations for Preventing Collisions at Sea), but the complex and high-risk environment at sea make it difficult to eliminate ship accidents [3]. Therefore, studies on maritime accidents will be helpful in guiding the management of maritime traffic safety and consequently reduce life and property loss [4].

The Taiwan Strait is a large channel between northern and southern China and is an important maritime passage connecting the Korean Peninsula, Japan, Southeast Asian countries, Hong Kong and Macao. With the steady increase in cargo throughput in Chinese ports, the number of ships sailing along the coast of China has also gradually increased. Taking the Taiwan Strait as an example, the number of 300 GT and above merchant ships passing through the Taiwan Strait every day during the three years from 2015 to 2017 is as high as 483 [5]. The increase in ship density and flow will inevitably lead to an increase in maritime traffic accident probability, among which ship collision accidents rank first among all kinds of accidents. The collision risk is a major indicator for navigators and surveillance operators to judge the collision danger between meeting ships [6], as well as the surveillance on shore plays an important role in preventing ship collision accidents [7]. Based on the historical statistical data, the number of collision accidents per unit time in a certain water area was considered to describe the regional collision risk by researchers, for example, the Formal Safety Assessment concept and Bayesian network method were used to evaluate the collision risk of ships in Yangtze River waters in China with real accident data [8]. Since historical ship collision accidents have the features of strong contingencies, small sample sizes and weak regularity, in general it is difficult to extract valuable information from historical data. So ship collision conflicts can be used as a substitute for ship collision accidents in characterizing the maritime traffic safety situation and have become an important part of methods that quantitatively study the traffic safety problem and its countermeasures. Therefore, it is of practical significance to carry out research on the analysis of collision conflicts and the prediction of future situations with the purpose of providing data support for early warning and future implementation of the maritime security strategy in China [9].

With the development of time series analysis, artificial intelligence, fuzzy logic, chaos theory, artificial neural network and statistical learning theory, a large number of methods have been proposed for maritime traffic accident prediction [10–15]. The performance of some classic time series prediction models fail to satisfy expectations due to the ship motion process complexity with nonlinearity and uncertainty in harsh climates [16]. Support vector machine (SVM), a novel type of machine learning algorithm, has a strong capacity for processing nonlinear data. Based on SVM, the support vector regression model (SVR model) is an effective method in solving regression problems [17]. Compared to the neural network model, the SVR model needs less training data. Even though SVR is an effective prediction method, non-stationary time series have a great impact on its prediction accuracy [18]. As a new type of SVM, the LSSVM greatly improves the convergence speed by solving the function estimation problem with the quadratic programming method [19], and it can be used for ship collision prediction research [20]. However, due to the intrinsic complexity of ship collision conflicts, it is difficult to describe the variation trend in ship collision conflicts. In order to construct a suitable prediction model, the original dataset features of ship accidents need to be considered. Since a ship accident depends on the climate, which has specific cycles such as year, month, and week, the ship collision conflict time series can be considered as a combination of subseries characterized by different frequencies. Each subseries corresponds to a range of frequencies, shows much more regularities and is predicted more accurately than the original ship collision conflict series. EMD, proposed by Huang [21], exhibits a strong generality in dealing with non-stationary data. This method can reflect the physical characteristics of the original time series signal without pre-set basis function. As a special signal processing technique, EMD can decompose a complex signal into a collection of IMFs and a residue, which are relatively stationary subseries and can be readily modelled [22, 23]. Discrete wavelet transform (DWT) is also a powerful method in dealing with non-stationary and nonlinear signals [24, 25]. But the processing procedure of DWT is not autoregressive and the decomposition accuracy is affected by the band-pass filters which are chose to decompose target signals. Wavelet basics function and decomposed layer also affect the decomposition results. Therefore, the decomposition accuracy of DWT is relatively lower than EMD, and EMD is used in the decomposition of ship collision conflict time series.

According to the above comprehensive analysis, in this work, an EMD-QPSO-LSSVM approach, which is a hybrid of empirical mode decomposition and quantum-behaved particle swarm optimization optimized least squares support vector machine model, is proposed to forecast ship collision conflicts. In the approach, the original ship collision conflict time series are decomposed into a collection of IMFs and a residue with EMD. Then, both the IMF components and the residue are used to establish the corresponding LSSVM models, where the key parameters of each LSSVM models are optimized by quantum- behaved PSO algorithm. Finally, the prediction values of the original ship collision conflict datasets are calculated by summing the forecasting values of every subseries. The effectiveness of the proposed model is verified using the real data from ship collision conflicts in the Taiwan Strait in 2014. The prediction results can, to some extent, provide a theoretical basis for the maritime department to develop an effective maritime management countermeasure and will be helpful in guiding the management of maritime traffic safety.

## 2. Objectives and contributions

Maritime transport plays an extremely important role in international trade and makes great contributions to national economic development. Shipping has long been regarded as a complex and high-risk activity, and maritime accidents often lead to serious damage, death, loss, injury or pollution, and may also have significant political, economic and environmental consequences. The collision risk is a major indicator for navigators and surveillance operators to judge the collision danger between meeting ships. In order to measure the collision risk, ship collision conflicts are used as an important index for measuring maritime traffic safety and maritime management. The objective of this study is to propose an efficient method to predict the future state by analysing the historical data of ship collision conflicts in the Taiwan Strait. The contribution of the work is the reference value for the administrative department in developing a maritime management countermeasure to reduce ship collision accidents.

## 3. Methodology

**Fig 1:**
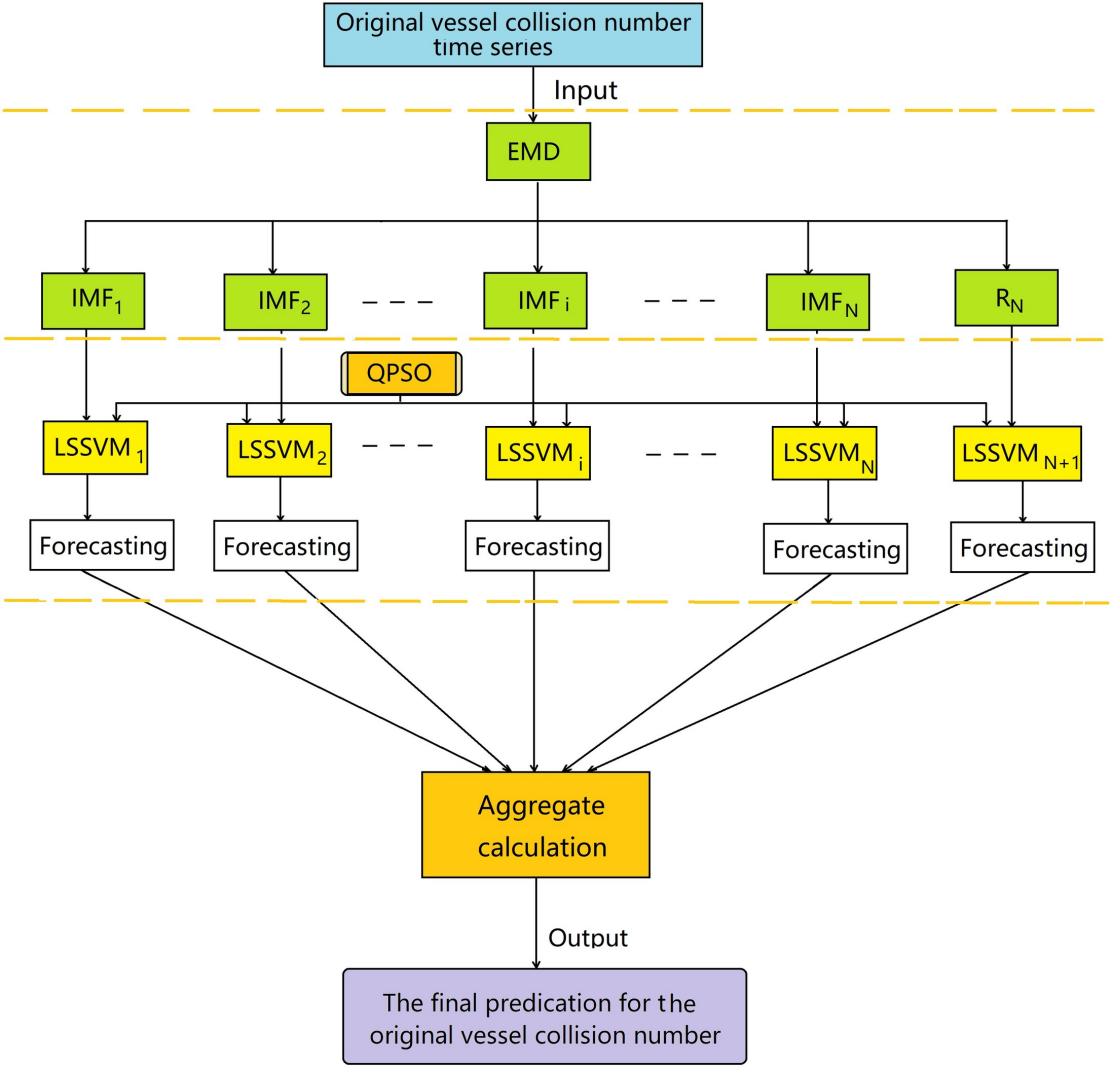
The flowchart of the EMD-QPSO-LSSVM method.

A hybrid of empirical mode decomposition and a least squares support vector machine model, named EMD-QPSO-LSSVM method, is proposed to forecast ship collision conflicts. The flowchart is shown in Fig 1. In the approach, the original ship collision conflict time series are decomposed into a collection of IMFs and a residue by EMD. Then, both the IMF components and the residue are used to establish the corresponding LSSVM models, where the key parameters of each LSSVM models are optimized by quantum-behaved particle swarm optimization algorithm. Finally, the prediction values of the original ship collision conflict datasets are calculated by summing the forecasting values of every subseries.

### 3.1 Empirical mode decomposition

Empirical mode decomposition method was first proposed by Huang [21]. In the prediction of non-stationary time series, EMD processing is very beneficial. This method can reflect the physical characteristics of the original time series signal without setting the basis function beforehand. The basic idea of empirical mode decomposition is that any set of signals consists of a limited number of intrinsic mode functions. According to the time scale characteristics of the data itself, the time series are decomposed step by step to extract IMF with different characteristic scales. Each IMF represents an intrinsic characteristic vibration form of the signal. The IMF needs to satisfy the following two basic conditions: i) The number of extrema and the number of zero-crossings should be equal or differ by one; ii) The average value of the upper envelope formed by the local maxima and the lower envelope formed by the local minima point should be zero.

Given an original ship collision conflict time series x(t), the EMD calculation can be described as follows:

$$x(t)=\sum_{k=1}^{n}{\mathrm{imf}}_{k}(t)+\mathrm{res}(t),$$ (1)

where imf_k_ is the k_th_ IMF and res(t) is the residue after the IMFs are derived. The empirical mode decomposition steps are as following:

Step 1. Find all the maximum and minimum points of original data sequence x(t), and fit all the maximum points with a cubic spline function. This curve is the upper envelope of data. All minimum points, similarly, are fitted with a cubic spline function to fit the lower envelope of data. Let m_1_(t) be the mean of the upper and the lower envelopes. By subtracting the mean value m_1_(t) from x(t), a new data sequence h_1_(t) is achieved.

$$h_{1}{(t)=x(t)‐m}_{1}(t).$$ (2)

If h_1_(t) does not satisfy the two basic requirements of IMF, the work above should be repeated with h_1_(t) as the original data until h_k_(t) meets the two requirements after k times. At this time h_k_(t) is imf_1_(t).

Step 2. A new data sequence x_2_(t) is achieved by subtracting IMF_1_(t) from the original data x(t).

$$x_{2}{(t)=x(t)‐\mathrm{imf}}_{1}(t).$$ (3)

Step 3. Repeat the above steps n times until the last data sequence x_n+1_(t) cannot be decomposed into IMF. This data sequence x_n+1_(t) is named the residue res(t) of the original data.

### 3.2 Quantum-behaved PSO-LSSVM

Least-squares-SVM is a very active artificial intelligence method and is widely applied in modelling and control problems [19, 26]. To optimize the LSSVM parameters, different algorithms were used in literature [20, 27–31]. Quantum-behaved particle swarm optimization algorithm is a kind of intelligent optimization algorithm developed on particle swarm optimization, and can be used to solve the nonlinear and complex optimization problems with the features of less control parameters, easily to set up, strong search capability and good global search ability [32, 33].

In this work, a modified QPSO algorithm is adopted [20], where the swarm updates the individuals’ positions in the following way:

$$\begin{array}{l} m_{best}[t]=\frac{1}{N}\sum_{i=1}^{N}p_{besti}[t]=\left(\frac{1}{N}\sum_{i=1}^{N}p_{besti1}[t],\cdots ,\frac{1}{N}\sum_{i=1}^{N}p_{bestiD}[t]\right),\\ p[t+1]=\varphi [t]\cdot p_{best}[t]+(1-\varphi [t])\cdot g_{best}[t],\\ x[t+1]=p[t]-\beta [t]\cdot |m_{best}[t]-x[t]|\ln(2u[t]), \end{array}$$ (4)

where φ[t],u[t] are random numbers in [0,1] at step *t*, *N* is the size of the swarm, *D* is the dimension of the particles, **g**_*best*_(*t*) is the entire swarm’s best known position, **p**_*besti*_[*t*] is the i*th* particle’s best known position, and **p**[*t*] is called a local attractor.

The inertia weight *β*[*t*] takes the following form

$$\beta [t]=\beta_{0}-\beta_{1}\chi [t]+\beta_{2}\lambda [t],$$ (5)

where *χ*[1] = *λ*[1] = 0 and

$$\chi [t]≜\frac{\mathrm{FIT}(g_{best}[t])}{\mathrm{FIT}(g_{best}[t-1])},\;\lambda [t]≜\frac{\mathrm{FIT}(g_{best}[t-1])}{\frac{1}{N}\sum_{i=1}^{N}\mathrm{FIT}(p_{best}{}_{i}[t-1])},2\leq t\leq t_{\max},$$

and *β*_0_,*β*_1_,*β*_2_ satisfy the constraints *β*_1_<*β*_0_ and *β*_0_+*β*_2_<1.78 as it was proved in [33] that as long as *β*[*t*]<1.78, the convergence of QPSO can be guaranteed.

For given a dataset $S=\{(x_{i}{,y}_{i}{)\}}_{i=1}^{N}$, where $x_{i}\in {\mathbb{R}}^{m}$ is input data in input space and $y_{i}\in \mathbb{R}$ is output value for given value of specific input variable, the LSSVM-based prediction model for the nonlinear function is

$$y(x)=\sum_{l=1}^{N}\alpha_{l}\cdot Kernal(x,x_{l})+b.$$ (6)

The parameters **α** = [*α*_1_,*α*_2_,⋯,*α*_*N*_]^*T*^ and *b* can be determined by

$$\left[\begin{array}{c} b\\ \alpha \end{array}\right]={\left[\begin{array}{cc} 0 & L^{T}\\ L & \Phi +\Gamma^{‐1} \end{array}\right]}^{-1}\left[\begin{array}{c} 0\\ Y \end{array}\right],$$ (7)

where **Y** = [*y*_1_,*y*_2_,⋯,*y*_*N*_]^*T*^, **L** = [1,1,⋯,1]^*T*^, Φ = (Φ_*ij*_)_*N*×*N*_ with general element Φ_*ij*_ = *φ*(**x**_*i*_)^*T*^*φ*(**x**_*j*_) = Kernal(**x**_*i*_,**x**_*j*_) and Γ = (Γ_*ij*_)_*N*×*N*_ with general element

$$\Gamma_{\mathrm{ij}}=\{\begin{array}{cc} \gamma_{i}≜\gamma_{0}\exp(\frac{i}{N}\rho ), & j=i,\\ 0, & j\neq i. \end{array}$$ (8)

**Fig 2:**
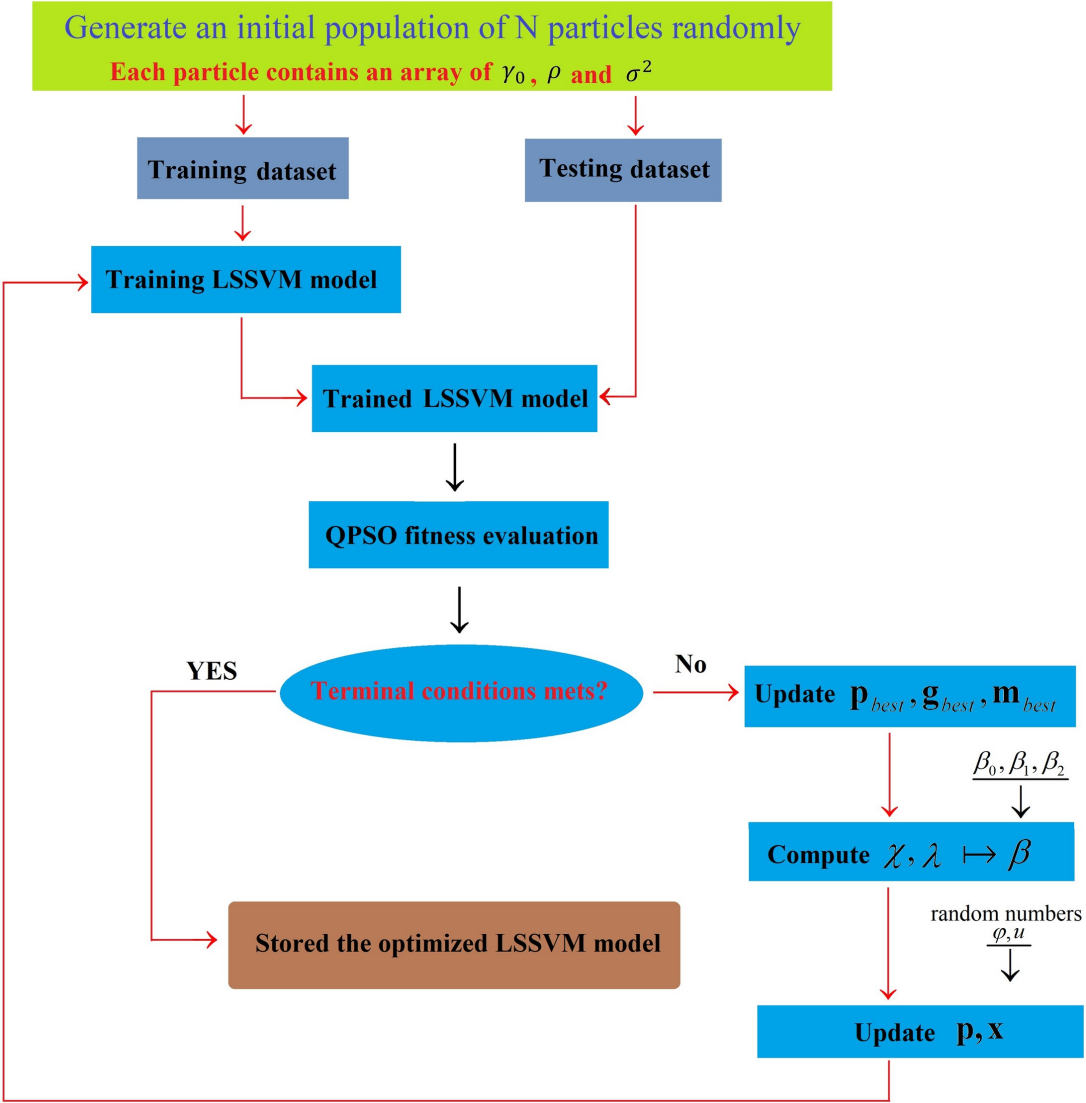
Flow chart of the parameters of the LSSVM model optimization by QPSO algorithm.

The kernel function Kernal(⋅) is chosen as the RBF kernel function, and the parameters γ_0_,ρ and *σ*^2^ are determined by QPSO algorithm. The flow chart of parameters adjustment QPSO-based is depicted in Fig 2. The optimization procedure has been repeated several times as attempts to reach the most probable global optimum of the fitness function.

## 4. Numerical simulations

### 4.1 Error measures

To assess the performance of the prediction models, three error measures are used for model comparison, i.e., the mean absolute error (MAE), the mean relative error (MRE), the mean square error (MSE) and the mean absolute percentage error (MAPE).

$$\begin{array}{l} e_{\mathrm{MAE}}=\frac{1}{N_{\mathrm{Pr}ed}}\sum_{j=1}^{N_{\mathrm{pred}}}\left|z^{P}(j)-z(j)\right|\\ e_{\mathrm{MAPE}}=\frac{1}{N_{\mathrm{Pr}ed}}\sum_{j=1}^{N_{\mathrm{pred}}}\left|\frac{z^{P}(j)-z(j)}{z(j)}\right|\\ e_{\mathrm{MSE}}=\frac{1}{N_{\mathrm{Pr}ed}}\sum_{j=1}^{N_{\mathrm{Pr}ed}}\sqrt{{\left(z^{p}(j)-z(j)\right)}^{2}}\\ e_{\mathrm{MAPE}}=\frac{1}{N_{\mathrm{Pr}ed}}\sum_{i=1}^{N_{\mathrm{Pr}ed}}\left|\frac{z^{p}(j)-z(j)}{z(j)}\right| \end{array}$$ (9)

where *N*_Pr*ed*_ is the prediction sample size and *z*(*j*) and *z*^*p*^(*j*) are the actual and forecast values during a time period, respectively.

### 4.2 Ship collision conflict datasets

To verify the validity of the proposed hybrid approach, ship collision conflict data from the Taiwan Strait are employed. The data consist of actual daily ship collision conflicts from 1999 to 2014 [34], and the verification is processed on the data in 2014, as presented in Table 1.

**Table 1:** Ship collision conflicts in the Taiwan Strait in 2014.

No.	Count	No.	Count	No.	Count	No.	Count	No.	Count	No.	Count	No.	Count
**1**	**198**	54	**149**	107	**241**	160	**193**	213	**250**	266	**240**	319	**211**
**2**	**211**	55	**139**	108	**151**	161	**201**	214	**169**	267	**303**	320	**323**
**3**	**246**	56	**156**	109	**199**	162	**193**	215	**158**	268	**291**	321	**241**
**4**	**182**	57	**117**	110	**220**	163	**195**	216	**157**	269	**262**	322	**301**
**5**	**217**	58	**115**	111	**219**	164	**196**	217	**226**	270	**283**	323	**423**
**6**	**206**	59	**131**	112	**222**	165	**198**	218	**266**	271	**343**	324	**376**
**7**	**255**	60	**134**	113	**172**	166	**160**	219	**429**	272	**271**	325	**377**
**8**	**201**	61	**177**	114	**261**	167	**370**	220	**386**	273	**217**	326	**288**
**9**	**233**	62	**195**	115	**268**	168	**212**	221	**300**	274	**304**	327	**286**
**10**	**275**	63	**186**	116	**270**	169	**250**	222	**261**	275	**276**	328	**321**
**11**	**224**	64	**108**	117	**219**	170	**282**	223	**394**	276	**223**	329	**301**
**12**	**222**	65	**139**	118	**149**	171	**238**	224	**265**	277	**320**	330	**302**
**13**	**170**	66	**176**	119	**168**	172	**175**	225	**200**	278	**201**	331	**264**
**14**	**188**	67	**186**	120	**180**	173	**202**	226	**193**	279	**142**	332	**284**
**15**	**258**	68	**162**	121	**290**	174	**248**	227	**297**	280	**296**	333	**264**
**16**	**217**	69	**149**	122	**246**	175	**225**	228	**253**	281	**295**	334	**275**
**17**	**229**	70	**163**	123	**260**	176	**162**	229	**230**	282	**303**	335	**346**
**18**	**185**	71	**130**	124	**180**	177	**227**	230	**252**	283	**181**	336	**250**
**19**	**231**	72	**131**	125	**176**	178	**196**	231	**294**	284	**384**	337	**275**
**20**	**211**	73	**149**	126	**308**	179	**178**	232	**221**	285	**401**	338	**264**
**21**	**152**	74	**131**	127	**242**	180	**152**	233	**293**	286	**196**	339	**249**
**22**	**201**	75	**154**	128	**285**	181	**210**	234	**269**	287	**301**	340	**291**
**23**	**187**	76	**135**	129	**184**	182	**223**	235	**271**	288	**200**	341	**260**
**24**	**163**	77	**130**	130	**185**	183	**225**	236	**194**	289	**244**	342	**320**
**25**	**151**	78	**128**	131	**181**	184	**223**	237	**262**	290	**317**	343	**319**
**26**	**128**	79	**146**	132	**210**	185	**233**	238	**299**	291	**250**	344	**332**
**27**	**163**	80	**117**	133	**138**	186	**240**	239	**254**	292	**304**	345	**276**
**28**	**140**	81	**180**	134	**143**	187	**276**	240	**230**	293	**266**	346	**267**
**29**	**167**	82	**191**	135	**218**	188	**185**	241	**261**	294	**302**	347	**295**
**30**	**159**	83	**177**	136	**201**	189	**228**	242	**324**	295	**223**	348	**278**
**31**	**178**	84	**158**	137	**111**	190	**147**	243	**223**	296	**260**	349	**290**
**32**	**102**	85	**140**	138	**174**	191	**170**	244	**215**	297	**226**	350	**248**
**33**	**128**	86	**122**	139	**196**	192	**283**	245	**297**	298	**269**	351	**309**
**34**	**127**	87	**152**	140	**175**	193	**276**	246	**300**	299	**268**	352	**294**
**35**	**110**	88	**131**	141	**178**	194	**210**	247	**264**	300	**179**	353	**254**
**36**	**135**	89	**136**	142	**220**	195	**233**	248	**277**	301	**282**	354	**230**
**37**	**121**	90	**120**	143	**143**	196	**240**	249	**270**	302	**222**	355	**320**
**38**	**102**	91	**128**	144	**174**	197	**227**	250	**267**	303	**265**	356	**314**
**39**	**111**	92	**176**	145	**161**	198	**163**	251	**240**	304	**203**	357	**348**
**40**	**100**	93	**169**	146	**156**	199	**207**	252	**383**	305	**299**	358	**294**
**41**	**105**	94	**241**	147	**229**	200	**231**	253	**302**	306	**201**	359	**290**
**42**	**101**	95	**253**	148	**178**	201	**292**	254	**277**	307	**287**	360	**298**
**43**	**103**	96	**176**	149	**186**	202	**191**	255	**188**	308	**444**	361	**278**
**44**	**100**	97	**255**	150	**171**	203	**151**	256	**230**	309	**392**	362	**245**
**45**	**100**	98	**158**	151	**180**	204	**130**	257	**180**	310	**318**	363	**257**
**46**	**150**	99	**187**	152	**201**	205	**133**	258	**139**	311	**242**	364	**210**
**47**	**140**	100	**212**	153	**171**	206	**274**	259	**282**	312	**274**	365	**186**
**48**	**127**	101	**203**	154	**192**	207	**287**	260	**256**	313	**291**		
**49**	**121**	102	**210**	155	**196**	208	**258**	261	**349**	314	**359**		
**50**	**152**	103	**115**	156	**150**	209	**294**	262	**193**	315	**352**		
**51**	**138**	104	**207**	157	**250**	210	**231**	263	**250**	316	**234**		
**52**	**133**	105	**227**	158	**229**	211	**207**	264	**190**	317	**239**		
**53**	**131**	106	**300**	159	**208**	212	**198**	265	**259**	318	**243**		

### 4.3 Data processing

The data processing follows the following steps:

*Step 1*: *EMD of the ship collision conflict time series*

**Fig 3:**
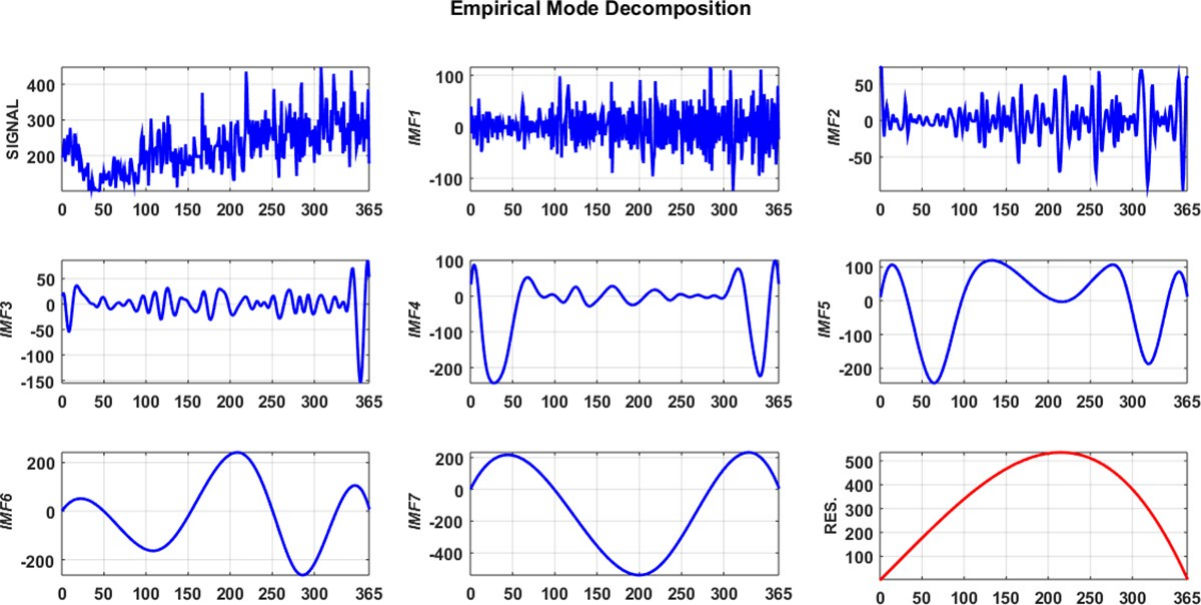
Schematic diagram of the EMD components.

Due to the intrinsic complexity of the original ship accident time series, the variation tendency is difficult to predict. To improve the prediction accuracy, EMD is used to decompose the original ship collision conflict time series **z** = (*z*_1_,*z*_2_,⋯,*z*_*T*_) with *T* = 365, which yields seven IMF components imf_*k*_ = (*z*_*k*1_,*z*_*k*2_,⋯,*z*_*kT*_)(*k* = 1,2,⋯,7) and a residue res = (*r*_1_,*r*_2_,⋯,*r*_*T*_), as illustrated in Fig 3.

*Step 2*: *Data normalization*

For the sake of expression, denote imf_*k*_ by **z**_*k*_ (*k* = 1,2,⋯,6) and res by **z**_7_ = {*z*_71_,*z*_72_,⋯,*z*_7*T*_}. Then normalize the sequence $z_{k}={\left\{z_{k1},z_{k2},\cdots ,z_{kT}\right\}}_{k=1}^{7}$ by Min–Max Normalization method [35] in the following form:

$${\bar{z}}_{ki}=\frac{z_{ki}-z_{k\min}}{z_{k\max}-z_{k\min}},i=1,2,\cdots ,T,k=1,\cdots ,7.$$

*Step 3*: *Data phase space reconstruction*

To sufficiently extract the useful information from time series ${\bar{z}}_{k}=\left({\bar{z}}_{k1},{\bar{z}}_{k2},\cdots ,{\bar{z}}_{kT}\right)$, the commonly used method is the phase space reconstruction (PSR) method in delay coordinates proposed by Packard et al. [36]. Theoretically speaking, a time series can sufficiently reconstruct an original dynamic system according to Takens [37]. From this procedure, time series ${\bar{z}}_{k}=\left({\bar{z}}_{k1},{\bar{z}}_{k2},\cdots ,{\bar{z}}_{kT}\right)$ can be reconstructed in a multidimensional phase space as follows:

$$x_{ki}=\left({\bar{z}}_{ki},{\bar{z}}_{k(i+\tau )},\cdots ,{\bar{z}}_{k(i+(m-1)\tau )}\right),y_{ki}={\bar{z}}_{k(i+m\tau )},i=1,\cdots ,T-m\tau ;k=1,\cdots ,7$$ (10)

where *τ* is the delay parameter and *m* is the embedding dimension. It is very important to select a suitable pair of embedding dimensions *m* and time delay *τ* when performing PSR [38–40]. There is no exact way to determine the values of *τ* and *m*, the result in [41] indicates that a larger value for *τ* than necessary should be selected to prevent system information from being ignored. Besides, according to the result in Brock et al [42], the appropriate values for embedded dimension *m* should be between 2 and 5. In the following simulations, the embedded dimension *m* is set equal to 4 and the time delay is assumed to be day to day.

### 4.4 Prediction by QPSO-LSSVM and representation

The data pair ${\left\{(x_{ki},y_{ki})\right\}}_{i=1}^{T_{1}}$ obtained in Eq (10) is used to train the QPSO-LSSVM and obtain an optimal parameter pair $\left(\gamma_{k0},\varrho_{k},\sigma_{k}^{2}\right)$, where *T*_1_ is the number of sample data in the training set. Then, the trained LSSVM is used to make a prediction

$${\bar{y}}_{kj}=LSSVM(x_{kj}),j=T_{1}+1,\cdots ,T-m\tau .$$ (11)

The final step is to carry out the reverse normalization on

$${\bar{z}}_{k}=\left({\bar{z}}_{k1},{\bar{z}}_{k2},\cdots ,{\bar{z}}_{kT_{1}},{\bar{y}}_{k(T_{1}+1)},\cdots ,{\bar{y}}_{k(T-m\tau )}\right),$$

which yields the sequence $z_{k}^{\prime }=\left(z_{k1}^{\prime },z_{k2}^{\prime },\cdots ,z_{kT}^{\prime }\right)$ and the prediction result is

$$z_{j}^{p}=\sum_{k=1}^{7}z_{kj}^{\prime },j=T_{1}+1,\cdots ,T.$$ (12)

### 4.5 Analysis of prediction results

To evaluate the prediction accuracy, the dataset is partitioned into a training dataset (90%) and a validation dataset (10%). The training dataset can be applied to establish the prediction model, and the validation dataset can be applied to validate the effectiveness of the model.

**Fig 4:**
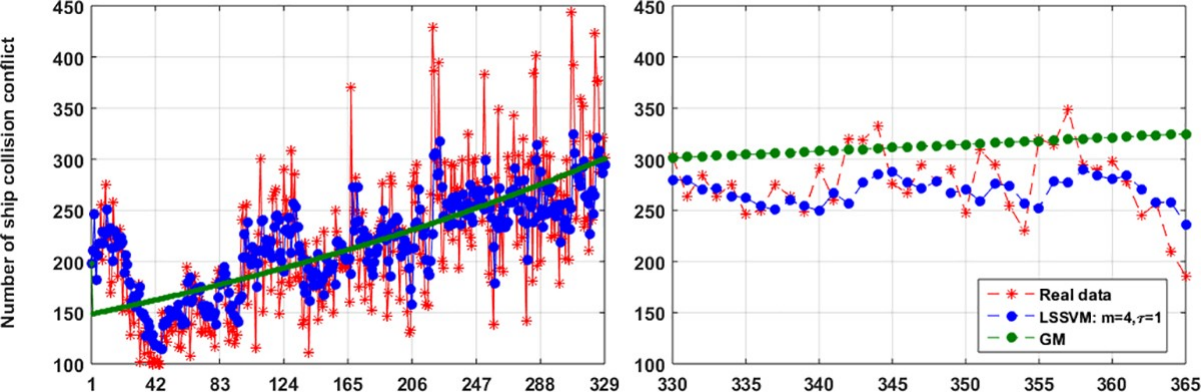
The prediction results of the GM and LSSVM for the ship collision conflicts dataset.

Grey model is easily set up, and the prediction result is presented in Fig 4. It can be observed that the prediction of GM is unsatisfied, and most of predictions are higher than the actual data. When LSSVM with key parameters *γ*_0_ = 10,*ρ*= 0,*σ* = 2 is applied, the prediction results for training dataset and testing dataset are shown in Fig 4. It is obvious that the performance of the LSSVM is better than that of the GM. The maximum error is about 24% and the mean square error is about 5, it is still not suitable for real applications.

In order to improve the prediction accuracy, QPSO algorithm is applied to search an optimal key parameters (*γ*_0_,*ϱ*,*σ*^2^). Here, the K-fold cross-validation is adopted to prevent the overfitting issue, and the training dataset is divided randomly into 9 folds, one of which was selected as the validation set each time for model selection, and the rest was used for model training. Table 2 illustrates the performance of LSSVM with 9-fold cross-validation.

**Table 2:** The result of 9-fold cross-validation.

Models	MSE on fold k	MSE on validation dataset
**LSSVM_1_**	4.68	4.62
**LSSVM_2_**	4.79	
**LSSVM_3_**	4.46	
**LSSVM_4_**	4.32	
**LSSVM_5_**	4.58	
**LSSVM_6_**	4.63	
**LSSVM_7_**	4.72	
**LSSVM_8_**	4.83	
**LSSVM_9_**	4.57	

**Fig 5:**
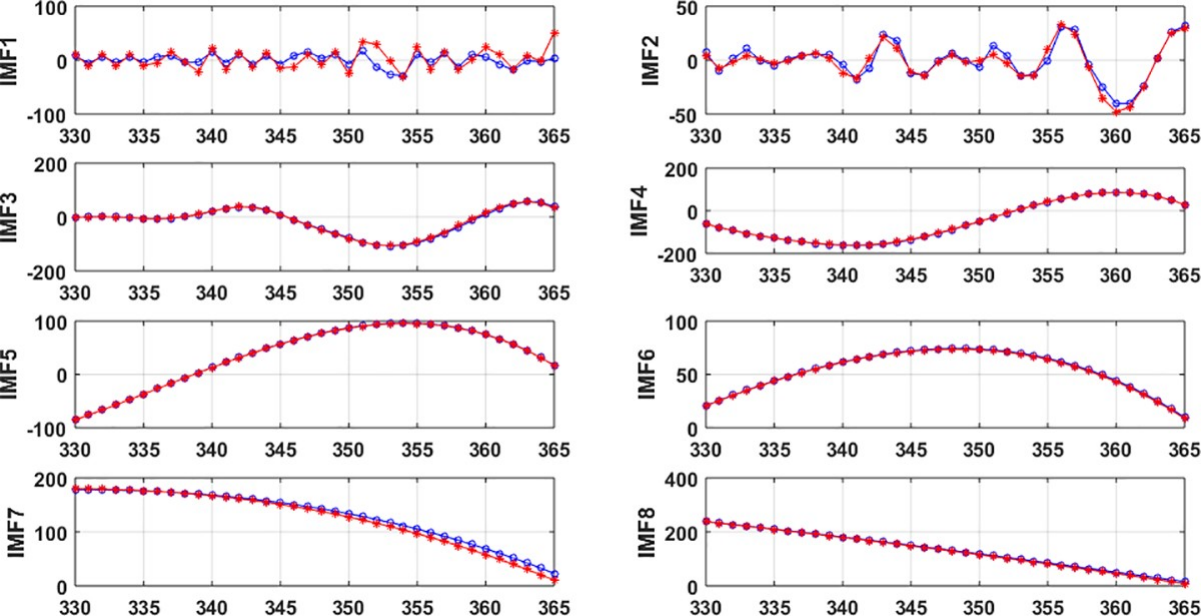
The prediction of the LSSVM for each IMF component.

Besides, due to the intrinsic complexity of ship collision, the regularity of the conflict time series is unobvious, and the prediction results directly from the original dataset is unsatisfied. Since a ship accident depends on the climate, which has specific cycles such as year, month, and week, the ship collision conflict time series can be considered as a combination of subseries characterized by different frequencies. Each subseries corresponds to a range of frequencies, shows much more regularities and is predicted more accurately than the original ship collision conflict series. The IMF components and residue by EMD is shown in Fig 3. The regularity of the latter five IMFs and residue is obviously stronger than the first two IMFs. By establishing different LSSVMs to the IMF components and residue, it can obtain a satisfied prediction results. The parameters of each LSSVM can be achieved by the flow chart of Fig 2. The prediction of the quantum-behaved PSO-LSSVM for each IMF component and residue are shown in Fig 5.

**Fig 6:**
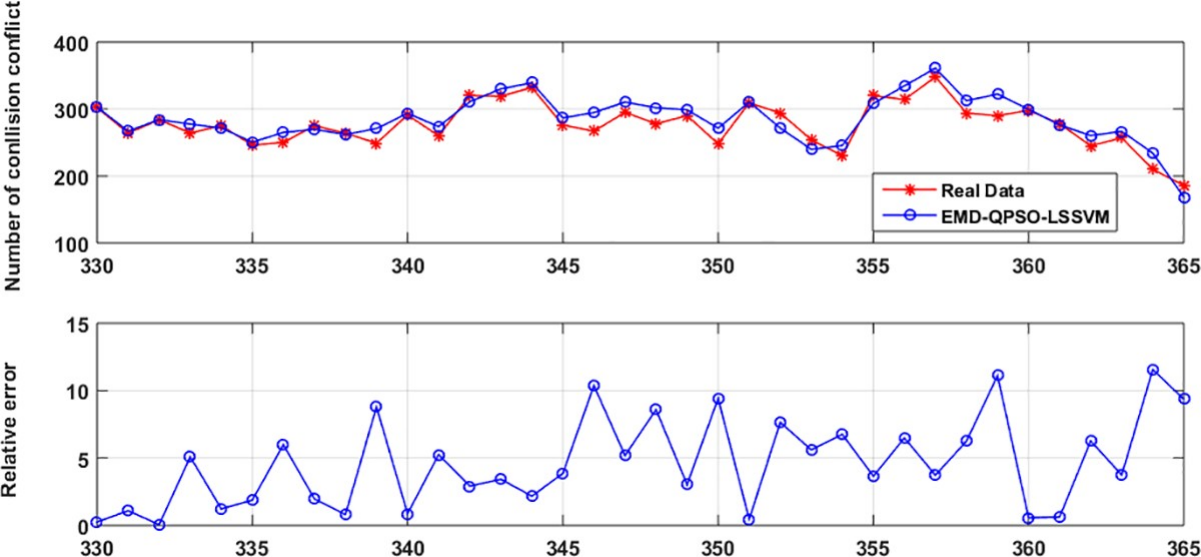
The final prediction of the EMD-LSSVM for the original ship collision conflict numbers.

The final prediction of the original ship collision conflict numbers are calculated by the sum of the prediction of each subseries, as shown in Fig 6. It can be seen that the prediction accuracy has been greatly improved. This indicates that the proposed method can be used for the prediction of ship collision conflicts as a substitute for ship collision accidents in characterizing the maritime traffic safety situation.

To evaluate the performance of the proposed method, the statistical test is carried out on the real data and the prediction result of EMD-QPSO-LSSVM, as shown in Table 3. The sig. is 0.212, which is greater than 0.05. Thus, the proposed method is suitable in predicting the ship collision conflict numbers.

**Table 3:** Statistical analysis on the performance of the proposed method.

Paired Samples Test								
				95% Confidence Interval of the Difference				
	Mean	Std. Deviation	Std. Error Mean	Low	Upper	t	df	Sig. (2-tailed)
**Real-Prediction**	-1.512	23.128	1.211	-3.893	0.868	-1.249	364	0.212

**Fig 7:**
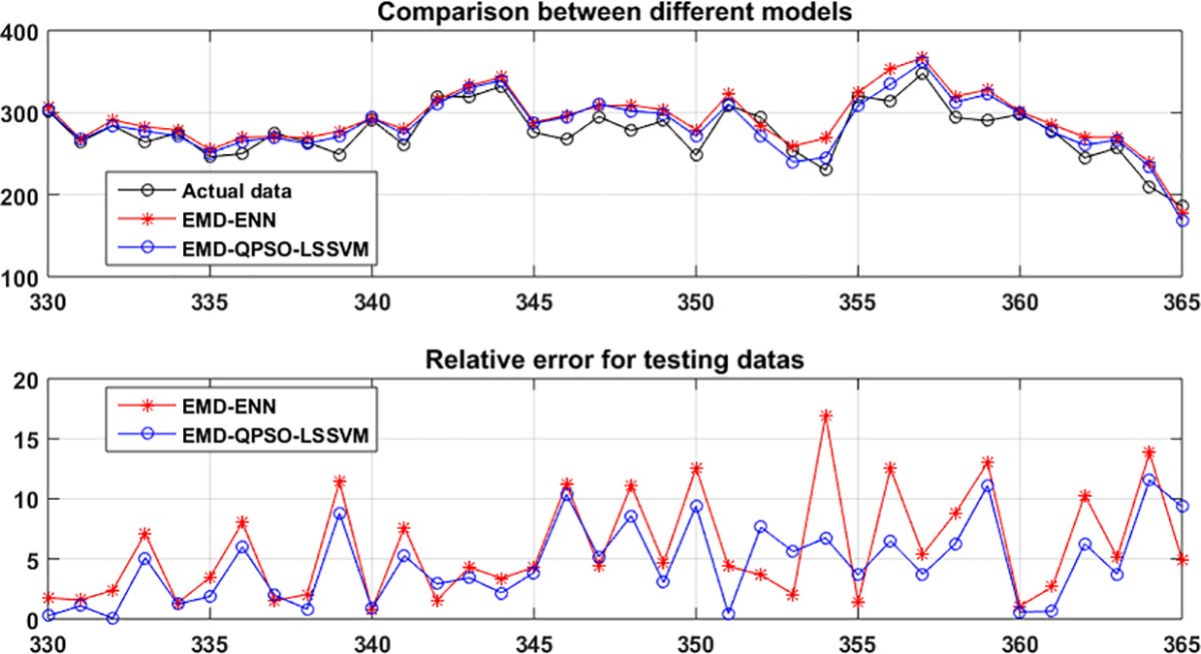
Comparison between EMD-QPSO -LSSVM and EMD-ENN for the original ship collision conflict numbers.

To verify the efficiency of the proposed method, it is compared with GM, Lasso Regression, Bayes Regression, LSSVR and EMD-ENN. The comparison results is shown in Fig 7 and Table 4, where ENN contains 15 neurons. It can be seen that the performance of EMD-QPSO-LSSVM is better than other methods. But it should also be pointed that EMD-ENN is also a suitable method for ship collision conflicts predication.

**Table 4:** Comparison between different methods.

error method	e_MAE_	e_MAPE_	e_MSE_	e_MSPE_	e_Max_
**GM(1,1)**	39.8732	19.9898	8.3172	131.6	74.642
**Lasso Regression**	31.0324	11.7076	6.4043	91.9915	48.8811
**Bayes Regression**	30.5444	11.4899	6.346	90.4829	47.9449
**LSSVR**	24.0912	8.6409	5.1562	66.2941	26.794
**EMD-ENN**	13.6143	5.0842	2.8862	39.1503	13.7046
**EMD-QPSO-LSSVM**	12.2919	4.6141	2.4832	34.1591	11.5604

Since there is no exact way to determine the choice of the embedded dimension, according to Brock et al [38], different simulations are carried out to show the influence of embedded dimension *m*, as shown in Table 5. For the ship collision conflicts, the embedded dimension can be set equal to 4 or 5 when the time delay is one.

**Table 5:** Influence of the embedded dimension on the error measures.

error m	e_MAE_	e_MAPE_	e_MSE_	e_MSPE_	e_Max_
**3**	16.7773	6.1433	3.5215	46.1189	15.6572
**4**	12.2919	4.6141	2.4832	34.1591	11.5604
**5**	12.5429	4.7489	2.4701	34.521	11.7386

## 5 Conclusion

The Taiwan Strait is a large channel between northern and southern China and is an important maritime passage connecting the Korean Peninsula, Japan, Southeast Asian countries, Hong Kong and Macao. The ship traffic flow is large, the navigation risk is high, and the daily average number of ship collision conflicts is approximately 220. The number of collision accidents per unit time in a certain water area can be used to describe the regional collision risk, which is the main index for evaluating maritime traffic safety and measuring maritime management. It is of great significance for maritime administrative authorities to formulate strategies to reduce ship collision accidents by predicting the occurrence of ship collision conflicts in the Taiwan Strait in a short period of time through historical collision conflicts. By considering the advantages of the empirical mode decomposition method, quantum-behaved PSO optimized least squares support vector machine, a hybrid of EMD and QPSO-LSSVM model, is proposed to forecast the ship collision conflicts. The original ship collision conflict time series are first decomposed into a collection of IMFs and a residue by EMD method. And then, both the IMF components and residue are applied to establish the corresponding LSSVM models, where the key parameters of the LSSVM are optimized by quantum-behaved PSO algorithm. Each subseries is predicted using the corresponding LSSVM. Finally, the prediction values of the original ship collision conflict datasets are calculated by the sum of the forecasting values of every subseries. The prediction results show that the EMD-QPSO-LSSVM is an efficient method and can be used in the forecasting of ship accidents.

## Supporting information

S1 File (DOCX)

S1 Data (RAR)
