# Supplementary material for: A study on ship collision conflict prediction in the Taiwan Strait using the EMD-based LSSVM method
Source: PLoS One. 2021 May 10;16(5):e0250948. doi: 10.1371/journal.pone.0250948 (PMC8109767; doi:10.1371/journal.pone.0250948)
Supplement: S1 File — (DOCX) [file pone.0250948.s001.docx]

*Research article*

Vessel collision conflict prediction in the Taiwan Strait using a hybrid of empirical mode decomposition and a least squares support vector machine model

Tian Chai^1^, Xue Han^1,*^

1. Navigation Institute, Jimei University, Xiamen, 361021, China

Correspondence should be addressed to imlmd@163.com

ABSTRACT Vessel collision accidents are the primary threat to traffic safety in the sea. Collision accidents can cause casualties and environmental pollution. This has attracted the attention of the maritime community. However, historical vessel collision accidents have contingencies, small sample sizes and weak regularities; hence, vessel collision conflicts can be used as a substitute for vessel collision accidents in characterizing the maritime traffic safety situation and have become an important part of methods that quantitatively study the traffic safety problem and its countermeasures. Vessel collision conflicts can be used as an important index for measuring traffic safety in the sea. In this work, an EMD-LSSVM approach, which is a hybrid of empirical mode decomposition (EMD) and a least squares support vector machine (LSSVM) model, is proposed to forecast vessel collision numbers. First, original vessel collision conflict time series are decomposed into a collection of intrinsic mode functions (IMFs) and a residue with EMD. Second, both the IMF components and residues are applied to establish the corresponding LSSVM models, where the key parameters of the LSSVM are optimized by an improved quantum-behaved particle swarm optimization (QPSO) algorithm. Then, each subseries is predicted with the corresponding LSSVM. Finally, the prediction values of the original vessel collision conflict datasets are calculated by the sum of the forecasting values of each subseries.

INDEX TERMS Forecast, Vessel collision conflicts, Taiwan Strait, Quantum-behaved particle swarm, Least squares support vector machine

I.  INTRODUCTION

Marine transportation, which accounts for more than 90% of the global freight volume, plays a key role in international trade. Ship navigation safety is a prerequisite for the normal operation of marine transportation systems. In recent years, with the steady increase in cargo throughput in Chinese ports, the number of ships sailing along the coast of China has also gradually increased. Taking the Taiwan Strait as an example, the number of 300 GT and above merchant ships passing through the Taiwan Strait every day during the three years from 2015 to 2017 is as high as 483**^[1]^**. The increase in ship density and flow will inevitably lead to an increase in maritime traffic accident probability, among which ship collision accidents rank first among all kinds of accidents. Once a ship collision accident occurs, it can cause casualties and heavy economic losses. However, historical vessel collision accidents have the advantages of strong contingencies, small sample sizes and weak regularity, and vessel collision conflicts can be used in place of vessel collision accidents to depict the situation of maritime traffic safety. Therefore, it is of practical significance to carry out research to predict vessel collision conflicts.

With the development of science and technology, accident management and control of vessel transportation systems has transferred gradually from vertical single data statistics and logic analysis of deducing the accident mechanism and formation model into the integrated analysis of transverse composite data and future crisis prevention, with the purpose of providing a basis to implement prediction and early warnings**^[2].^**

Marine traffic engineering is a complex system engineering problem that has certain randomness and contingencies due to the influence of the navigation environment, hydrometeorology, crew capacity and undefined ship state. In view of this, vessel collision conflicts can be used as an important index for measuring the traffic safety of the sea. Therefore, the analysis of collision conflicts and the prediction of future situations can provide data support for the future implementation of the maritime safety strategy in China.

The widely applied vessel collision prediction methods mainly include the regression analysis method **^[3]^**, fractal theory **^[4]^**, the grey system model **^[5-11]^**, and the Markov model **^[12-13]^**. Regression analysis is a statistical inference method used to study the relevant relationships among the phenomena (variables). The advantage of the regression analysis method is its ability to synthesize various factors of a vessel traffic system, but it requires a large amount of system data. The grey system model takes an uncertain system characterized by incomplete information as the research object, and through grey information processing, it seeks the laws in the system evolution process and then reduces the uncertainty of the internal information in the system. Compared with the regression analysis method, the grey system model can obtain more accurate short-term predictions with less data, but it is only suitable for modelling the series data with exponential and approximate exponential rules and describes the monotonous changing process.

Support vector machines (SVMs) are a new type of neural network that follow the principle of structural risk minimization, are suitable for small samples and nonlinear problems, and can effectively avoid dimensional disasters. In the case of a small dataset, an SVM can describe the nonlinear and random characteristics of vessel accidents. As a new type of SVM, the LSSVM greatly improves the convergence speed by solving the function estimation problem with the quadratic programming method **^[14]^**, which is more suitable for vessel collision prediction research. The performance of the LSSVM depends on the choice of parameters, which are determined in general by the cross-validation method. However, the limitations of the cross-validation method itself will affect the learning and generalization ability of the LSSVM. The quantum-behaved particle swarm optimization (QPSO) algorithm developed on particle swarm optimization **^[15]^** is a kind of intelligent optimization algorithm with the outstanding features of QPSO, including fewer control parameters, ease of set up, strong search capability and good global search ability, and it can be used to solve nonlinear and complex optimization problems.

When using models for vessel collision conflict forecasting, the original vessel collision conflict time series are usually applied directly to build prediction models. However, due to the intrinsic complexity of vessel collision conflicts, it is difficult to describe the variation trend in vessel collision conflicts. To construct a suitable prediction model, the original dataset features of vessel accidents need to be considered. Since a vessel accident depends on the climate, which has specific cycles such as year, month, and week., the vessel collision conflict time series can be considered a combination of subseries characterized by different frequencies. Each subseries corresponds to a range of frequencies, shows much more regularities and is predicted more accurately than the original vessel collision conflict series. As a special signal processing technique, empirical mode decomposition (EMD) can decompose a complex signal into a collection of intrinsic mode functions (IMFs) and a residue, which are relatively stationary subseries and can be readily modelled **^[16-18]^**. As an application, Wang et al **^[18]^** used the EMD method to decompose the wind speed time series and made a prediction with the Elman neural network.

In this work, an EMD-LSSVM approach, which is a hybrid of empirical mode decomposition and a least squares support vector machine model, is proposed to forecast vessel collision conflicts. In the approach, the original vessel collision conflict time series are decomposed into a collection of IMFs and a residue with EMD. Then, both the IMF components and the residue are used to establish the corresponding LSSVM models, where the key parameters of each LSSVM models are optimized by the QPSO algorithm. Finally, the prediction values of the original vessel collision conflict datasets are calculated by summing the forecasting values of every subseries. The effectiveness of the proposed model is verified using the real data from vessel collision conflicts in the Taiwan Strait in 2014. The prediction results can, to some extent, provide a theoretical basis for the maritime department to develop an effective maritime management countermeasure.

II. OBJECTIVES AND CONTRIBUTIONS

Maritime transport plays an extremely important role in international trade and makes great contributions to national economic development. Vessel collision conflicts are an important index for measuring maritime traffic safety and maritime management. The objective of this study is to predict the future state by analysing the historical data of vessel collision conflicts. The contribution of this study it is a method that provides an efficient way to predict the number of vessel collision conflicts in the Taiwan Strait, and it is helpful to the administrative department in developing a maritime management countermeasure to reduce vessel collision accidents.

III. METHODOLOGY

In this work, an EMD-LSSVM approach, which is a hybrid of empirical mode decomposition (EMD) and a least squares support vector machine (LSSVM) model, is proposed to forecast vessel collision conflicts. First, the original vessel collision conflict time series are decomposed into a collection of intrinsic mode functions (IMFs) and a residue with EMD. Second, both the IMF components and residue are applied to establish the corresponding LSSVM models, where the key parameters of the LSSVM models are optimized by an improved quantum-behaved particle swarm optimization (IQPSO) algorithm. Then, each subseries is predicted using the corresponding LSSVM. Finally, the prediction values of the original vessel collision conflict datasets are calculated by the sum of the forecasting values of every subseries.

We followed the description proposed by Wang et al. **^[18]^**, and the flowchart is shown in Fig. 1. The methods used in the EMD-LSSVM approach are briefly stated in the following.

*EMPIRICAL MODE DECOMPOSITION (EMD)*

The foundation of the EMD method is to decompose a complicated signal into a sum of several intrinsic mode functions (IMFs) and a residue, which are relatively stationary subseries and can be readily modelled. In the EMD method, a function is called an IMF if it satisfies the following two conditions: (i) in the whole dataset, the sum of the extremes and the sum of the zero crossings must be equal or differ at most by one, and (ii) the average of the envelope, which is defined by the local maxima and minima, must be zero at any point **^[17, 18]^**. Given an original vessel collision conflict time series, the EMD calculation can be described as follows:

$x(t)=\sum_{k=1}^{N} f_{k}(t)+R_{N}(t)$,

where $f_{k}(t)(k=1,2,\cdots,N)$ represents different IMFs and $R_{N}(t)$ is the residue after the IMFs are derived.


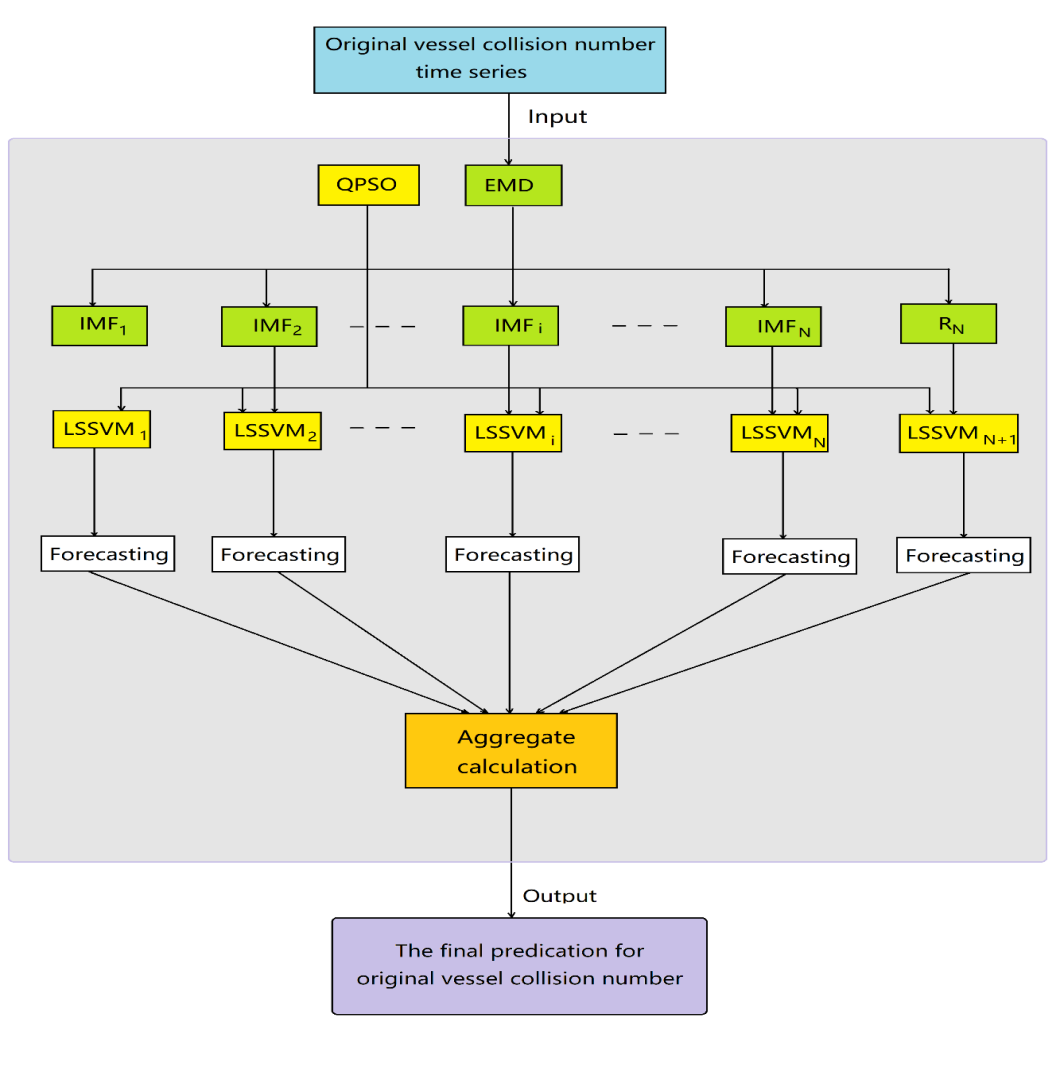


Fig. 1 The flowchart of the EMD-QPSO-LSSVM method

For convenience, the sifting process is described as follows:

*Step 1*: Identify all the local extremes of the original vessel collision conflict time series $\{x(t)\}$;

*Step 2*: Calculate its upper envelope $\{x_{1}(t)\}$, which can be derived by connecting all the local maxima using a cubic spline line. Similarly, we can obtain the lower envelope $\{x_{2}(t)\}$;

*Step 3*: Apply the upper and lower envelopes to calculate the mean envelope $\{h(t)\}$, which can be represented as follows:

$$h(t):=\frac{x_{1}(t)+x_{2}(t)}{2}$$

*Step 4*: Find the IMF. Let $m(t):=x(t)-h(t)$. If $m(t)$ is an IMF, then set $f(t)=m(t)$ and replace $x(t)$ with residual $r(t)=x(t)-f(t)$. Otherwise, replace $x(t)$ with $m(t)$, and repeat *Steps 2-3* until residual $R_{N}(t)$ is monotonous.

*Step 5*: Repeat *steps 1-4* until all the IMFs are found.

*LSSVM regression algorithm*

The LSSVM takes the regularization theory and structural risk minimization as the basis, greatly reducing the computational complexity by changing the quadratic programming problem in a standard SVM into solving linear equations. At present, the LSSVM is a very active artificial intelligence method and is widely applied in modelling and control problems.

For a training set $S=\{(\mathbf{x}_{i},y_{i})\}_{i=1}^{N}$, where , building the LSSVM model is equivalent to solving the following constrained optimization problems:

where $w$ and $b$ are the coefficient and the constant term of the linear estimation function in the high dimensional feature space, respectively; $\gamma$ is the penalty factor; $e_{i}$ is the fitting error for individual $i$; and is a mapping from the data space to a higher dimensional feature space.

The above optimization problem can be converted to a dual problem through the Lagrange multiplier. The conditions for the optimal solutions are

$\left[\begin{matrix} b \\ \alpha\end{matrix}\right]=\left[\begin{matrix} 0 & \mathbf{L}^{T} \\ \mathbf{L} & \Phi+\Gamma^{\text{-1}} \end{matrix}\right]^{\text{-1}}\left[\begin{matrix} 0 \\ \mathbf{Y} \end{matrix}\right]$, (1)

where $\alpha=[\alpha_{1},\alpha_{2},\cdots,\alpha_{N}]^{T}$ is the Lagrange multiplier, $\mathbf{Y}=[y_{1},y_{2},\cdots,y_{N}]^{T}$, $\mathbf{L}=[1,1,\cdots,1]^{T}$, and $\Phi=(\Phi_{ij})_{n\times n}$ is an *N-*order square matrix with general element . Here, different weights $\gamma_{i}$ are assigned to the $i_{\text{th}}$ data error; thus, $\Gamma$ satisfies

(2)

where $\gamma_{0}$ and are two positive constants.

The LSSVM-based prediction model for the nonlinear function is

$y(\mathbf{x})=\sum_{l=1}^{N} \alpha_{l}\cdot Kernal(\mathbf{x},\mathbf{x}_{l})+b$. (3)

The kernel function $Kernal(\cdot)$ is chosen as the RBF kernel function since the generalization ability of the RBF is stronger.

*QPSO ALGORITHM*

Quantum-behaved particle swarm optimization (QPSO) was proposed by Sun et al**^[15]^** and was inspired by the basic theory of quantum physics, which mainly adopts the expression characteristics of the superposition of quantum theory and probability features.

In the QPSO algorithm, the swarm updates the individuals’ positions in the following way:

(4a)

(4b)

$\mathbf{x}[ ]=\mathbf{p}[ ]+\delta(u[ ])\cdot\ln((u[ ])^{\beta[ ]\cdot|\mathbf{m}_{best}[ ]-\mathbf{x}[ ]|})$ (4c)

where are random numbers in [0, 1], *N* is the size of the swarm, *D* is the dimension of the particles, and $\mathbf{p}[ ]$ is called a local attractor. Function $\delta(u[ ])$ satisfies

$\delta(u[ ])=\left\{\begin{matrix} -1, & u[ ]\leq0.5, \\ 1, & u[ ]>0.5. \end{matrix}\right.$ (5)

Note that $u[ ]$ in Eq. (5) is a random number in [0, 1] that obeys a uniform distribution and $\text{E}\left(u[]\right)=0.5$, which indicates that when *u*[]=0.5, the position of particle **x**[] should be assigned at a local attractor **p**[]. However, from Eq. (5), $\delta(0.5)=-1$, and thus $\mathbf{x}[ ]$ is not assigned to $\mathbf{p}[ ]$ when $u[]=0.5$. Based on this consideration, a modification of Eq. (4c) is made; i.e.,

$\mathbf{x}[ ]=\mathbf{p}[ ]-\beta[ ]\cdot|\mathbf{m}_{best}[ ]-\mathbf{x}[ ]|\ln(2u[ ])$. (4c’)

In this work, a dynamically adjusting inertia weight $\beta[ ]$ is adopted. Let FIT denote the fitness function in a minimization problem. Set $\chi[1]=\lambda[1]=0$. For $n=2,3,\cdots,n_{\max}$, define

. (5)

Notably, $0\leq\chi[n],\lambda[n]\leq1$, where $\chi[n]$ reflects the evolution speed of the quantum particle swarm and $\lambda[n]$ reflects all the particle aggregation degrees. A dynamically adjusting inertia weight $\beta[ ]$ is adapted, and it takes the following form:

$\beta[ ]=\beta_{0}-\beta_{1}\chi[ ]+\beta_{2}\lambda[ ]$, (6)

where $\beta_{0}$ is the initial weight and, in general, it is assumed that $\beta_{0}=1$; $\beta_{1}$ and $\beta_{2}$ are the weights of $\chi[n]$ and $\lambda[n]$. Since $\chi[n]$ and $\lambda[n]$ are both dependent on the iteration step, then $\beta[ ]$ in Eq. (5) is also dependent on iteration step but in an indirect way. Since it was proven that as long as $\beta[ ]<1.78$, the convergence of QPSO can be guaranteed[14]; thus, it is assumed that $\beta_{i}(i=0,1,2)$ satisfies the constraints, $\beta_{1}<\beta_{0}$ and $\beta_{0}+\beta_{2}<1.78$.

When the RBF is chosen as the kernel function; i.e.,

$Kernal(\mathbf{x},\mathbf{x}')=\exp\left(-||\mathbf{x}-\mathbf{x}'||/2\sigma^{2}\right)$. (7)

The parameters to be optimized are the regularization parameter $\gamma_{0}$, in Eq. (3) and kernel parameter $\sigma$ in Eq. (7). The selection of the parameters has important effects on the learning and generalization ability of the model. In this work, QPSO is applied to optimize the parameters of the LSSVM.

For a training set $S=\{(\mathbf{x}_{i},y_{i})\}_{i=1}^{N}$, the detailed step of the IQPSO-LSSVM is described as follows:

*Step 1*: Initialize all the parameters of QPSO: swarm size *N*, maximum iterative step $n_{\max}$, coefficient $\beta_{i}(i=0,1,2)$ of the contraction-expansion factor, and initial positions of the particles.

*Step 2*: Make a prediction on the learning sample by applying the LSSVM corresponding to each particle, obtain the prediction error of the particle's current position and take it as the fitness value of each particle. Comparing the current fitness value with the particle's best fitness value, if the current fitness value is optimal, then update the particle's best position with the current position. Otherwise, keep the particle's best position.

*Step 3*: Compare the fitness value of each particle's best position with that of the swarm's best position. If there exists some particle's position that is more optimal than the swarm's best position, then update the swarm's position by that particle's position. Otherwise, keep the swarm's best position.

*Step 4*: Compute the inertia weight according to Eq. (2) and update the particle's position according to Eq. (4b) and Eq. (4c).

*Step 5*: Check whether it satisfies the conditions to end the optimization. If the ending condition is satisfied, then the process is ended, and the optimal solution is obtained. Otherwise, go to *Step 2*, and continue a new search process.

IV. NUMERICAL SIMULATIONS

*ERROR MEASURES*

To assess the performance of the prediction models, three error measures are used for model comparison, i.e., the mean absolute error (MAE), the mean relative error (MRE), the mean square error (MSE) and the mean absolute percentage error (MAPE).

$\text{e}_{\text{MAE}}=\frac{1}{N_{\Pr ed}}\sum_{j=1}^{N_{\text{pred}}} \left|z^{P}(j)-z(j)\right|$ (8a)

$\text{e}_{\text{MAPE}}=\frac{1}{N_{\Pr ed}}\sum_{j=1}^{N_{\text{pred}}} \left|\frac{z^{P}(j)-z(j)}{z(j)}\right|$ (8b)

$\text{e}_{\text{MSE}}=\frac{1}{N_{\Pr ed}}\sum_{j=1}^{N_{\Pr ed}} \sqrt{\left(z^{p}(j)-z(j)\right)^{2}}$ (8c)

$\text{e}_{\text{MAPE}}=\frac{1}{N_{\Pr ed}}\sum_{i=1}^{N_{\Pr ed}} \left|\frac{z^{p}(j)-z(j)}{z(j)}\right|$ (8d)

where $N_{\Pr ed}$ is the prediction sample size and $z(j)$ and $z^{p}(j)$ are the actual and forecast values during a time period, respectively.

*VESSEL collision CONFLICT datasets*

To verify the validity of the proposed hybrid approach, vessel collision conflict data from the Taiwan Strait are employed. These data consist of actual daily vessel collision conflicts in 2014**^[2]^**, which are given in Table 1, and the time series is shown in Fig. 2, where the numbers of vessel collision conflicts $\mathbf{z}=\{z_{1},z_{2},\cdots,z_{365}\}$ are presented in blue and the average numbers of vessel collision conflicts are presented in red.

Table 1 Vessel collision conflicts in the Taiwan Strait in 2014

| **No.** | **Count** | **No.** | **Count** | **No.** | **Count** | **No.** | **Count** | **No.** | **Count** | **No.** | **Count** | **No.** | **Count** |
| --- | --- | --- | --- | --- | --- | --- | --- | --- | --- | --- | --- | --- | --- |
| 1 | **198** | 54 | **149** | 107 | **241** | 160 | **193** | 213 | **250** | 266 | **240** | 319 | **211** |
| 2 | **211** | 55 | **139** | 108 | **151** | 161 | **201** | 214 | **169** | 267 | **303** | 320 | **323** |
| 3 | **246** | 56 | **156** | 109 | **199** | 162 | **193** | 215 | **158** | 268 | **291** | 321 | **241** |
| 4 | **182** | 57 | **117** | 110 | **220** | 163 | **195** | 216 | **157** | 269 | **262** | 322 | **301** |
| 5 | **217** | 58 | **115** | 111 | **219** | 164 | **196** | 217 | **226** | 270 | **283** | 323 | **423** |
| 6 | **206** | 59 | **131** | 112 | **222** | 165 | **198** | 218 | **266** | 271 | **343** | 324 | **376** |
| 7 | **255** | 60 | **134** | 113 | **172** | 166 | **160** | 219 | **429** | 272 | **271** | 325 | **377** |
| 8 | **201** | 61 | **177** | 114 | **261** | 167 | **370** | 220 | **386** | 273 | **217** | 326 | **288** |
| 9 | **233** | 62 | **195** | 115 | **268** | 168 | **212** | 221 | **300** | 274 | **304** | 327 | **286** |
| 10 | **275** | 63 | **186** | 116 | **270** | 169 | **250** | 222 | **261** | 275 | **276** | 328 | **321** |
| 11 | **224** | 64 | **108** | 117 | **219** | 170 | **282** | 223 | **394** | 276 | **223** | 329 | **301** |
| 12 | **222** | 65 | **139** | 118 | **149** | 171 | **238** | 224 | **265** | 277 | **320** | 330 | **302** |
| 13 | **170** | 66 | **176** | 119 | **168** | 172 | **175** | 225 | **200** | 278 | **201** | 331 | **264** |
| 14 | **188** | 67 | **186** | 120 | **180** | 173 | **202** | 226 | **193** | 279 | **142** | 332 | **284** |
| 15 | **258** | 68 | **162** | 121 | **290** | 174 | **248** | 227 | **297** | 280 | **296** | 333 | **332** |
| 16 | **217** | 69 | **149** | 122 | **246** | 175 | **225** | 228 | **253** | 281 | **295** | 334 | **205** |
| 17 | **229** | 70 | **163** | 123 | **260** | 176 | **162** | 229 | **230** | 282 | **303** | 335 | **346** |
| 18 | **185** | 71 | **130** | 124 | **180** | 177 | **227** | 230 | **252** | 283 | **181** | 336 | **211** |
| 19 | **231** | 72 | **131** | 125 | **176** | 178 | **196** | 231 | **294** | 284 | **384** | 337 | **275** |
| 20 | **211** | 73 | **149** | 126 | **308** | 179 | **178** | 232 | **221** | 285 | **401** | 338 | **264** |
| 21 | **152** | 74 | **131** | 127 | **242** | 180 | **152** | 233 | **293** | 286 | **196** | 339 | **229** |
| 22 | **201** | 75 | **154** | 128 | **285** | 181 | **210** | 234 | **269** | 287 | **301** | 340 | **291** |
| 23 | **187** | 76 | **135** | 129 | **184** | 182 | **223** | 235 | **271** | 288 | **200** | 341 | **260** |
| 24 | **163** | 77 | **130** | 130 | **185** | 183 | **225** | 236 | **194** | 289 | **244** | 342 | **169** |
| 25 | **151** | 78 | **128** | 131 | **181** | 184 | **223** | 237 | **262** | 290 | **317** | 343 | **319** |
| 26 | **128** | 79 | **146** | 132 | **210** | 185 | **233** | 238 | **299** | 291 | **250** | 344 | **432** |
| 27 | **163** | 80 | **117** | 133 | **138** | 186 | **240** | 239 | **254** | 292 | **304** | 345 | **376** |
| 28 | **140** | 81 | **180** | 134 | **143** | 187 | **276** | 240 | **230** | 293 | **266** | 346 | **267** |
| 29 | **167** | 82 | **191** | 135 | **218** | 188 | **185** | 241 | **261** | 294 | **302** | 347 | **295** |
| 30 | **159** | 83 | **177** | 136 | **201** | 189 | **228** | 242 | **324** | 295 | **223** | 348 | **378** |
| 31 | **178** | 84 | **158** | 137 | **111** | 190 | **147** | 243 | **223** | 296 | **260** | 349 | **290** |
| 32 | **102** | 85 | **140** | 138 | **174** | 191 | **170** | 244 | **215** | 297 | **226** | 350 | **218** |
| 33 | **128** | 86 | **122** | 139 | **196** | 192 | **283** | 245 | **297** | 298 | **269** | 351 | **229** |
| 34 | **127** | 87 | **152** | 140 | **175** | 193 | **276** | 246 | **300** | 299 | **268** | 352 | **315** |
| 35 | **110** | 88 | **131** | 141 | **178** | 194 | **210** | 247 | **264** | 300 | **179** | 353 | **284** |
| 36 | **135** | 89 | **136** | 142 | **220** | 195 | **233** | 248 | **277** | 301 | **282** | 354 | **230** |
| 37 | **121** | 90 | **120** | 143 | **143** | 196 | **240** | 249 | **270** | 302 | **222** | 355 | **270** |
| 38 | **102** | 91 | **128** | 144 | **174** | 197 | **227** | 250 | **267** | 303 | **265** | 356 | **274** |
| 39 | **111** | 92 | **176** | 145 | **161** | 198 | **163** | 251 | **240** | 304 | **203** | 357 | **348** |
| 40 | **100** | 93 | **169** | 146 | **156** | 199 | **207** | 252 | **383** | 305 | **299** | 358 | **254** |
| 41 | **105** | 94 | **241** | 147 | **229** | 200 | **231** | 253 | **302** | 306 | **201** | 359 | **212** |
| 42 | **101** | 95 | **253** | 148 | **178** | 201 | **292** | 254 | **277** | 307 | **287** | 360 | **298** |
| 43 | **103** | 96 | **176** | 149 | **186** | 202 | **191** | 255 | **188** | 308 | **444** | 361 | **278** |
| 44 | **100** | 97 | **255** | 150 | **171** | 203 | **151** | 256 | **230** | 309 | **392** | 362 | **245** |
| 45 | **100** | 98 | **158** | 151 | **180** | 204 | **130** | 257 | **180** | 310 | **318** | 363 | **313** |
| 46 | **150** | 99 | **187** | 152 | **201** | 205 | **133** | 258 | **139** | 311 | **242** | 364 | **382** |
| 47 | **140** | 100 | **212** | 153 | **171** | 206 | **274** | 259 | **282** | 312 | **274** | 365 | **186** |
| 48 | **127** | 101 | **203** | 154 | **192** | 207 | **287** | 260 | **256** | 313 | **291** |  |  |
| 49 | **121** | 102 | **210** | 155 | **196** | 208 | **258** | 261 | **349** | 314 | **359** |  |  |
| 50 | **152** | 103 | **115** | 156 | **150** | 209 | **294** | 262 | **193** | 315 | **352** |  |  |
| 51 | **138** | 104 | **207** | 157 | **250** | 210 | **231** | 263 | **250** | 316 | **234** |  |  |
| 52 | **133** | 105 | **227** | 158 | **229** | 211 | **207** | 264 | **190** | 317 | **239** |  |  |
| 53 | **131** | 106 | **300** | 159 | **208** | 212 | **198** | 265 | **259** | 318 | **243** |  |  |


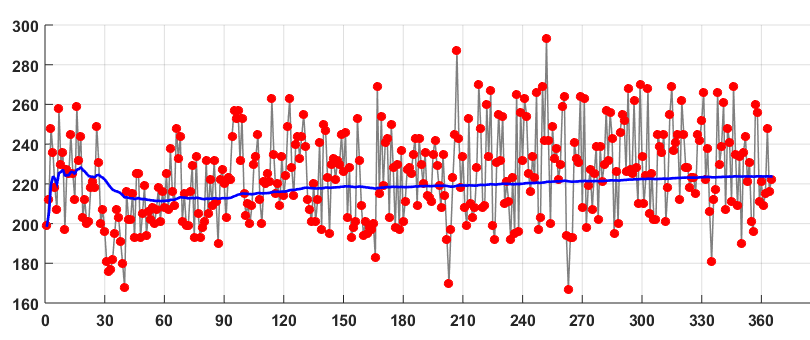


Fig. 2 Data on vessel collision conflicts in the Taiwan Strait in 2014

*DATA PREPROCESSING*

*Step 1 EMD of the vessel collision conflict sequence*

Due to the intrinsic complexity of the original vessel accident time series, the variation tendency is difficult to describe. To improve the prediction accuracy, EMD is applied to decompose the original vessel collision conflict sequence$\mathbf{z}=\{z_{1},z_{2},\cdots,z_{T}\}$, and the decomposition results ($\mathbf{z}_{k}=\{z_{k1},z_{k2},\cdots,z_{kT}\}_{k=1}^{N_{1}\text{+1}}$) are shown in Fig. 3.


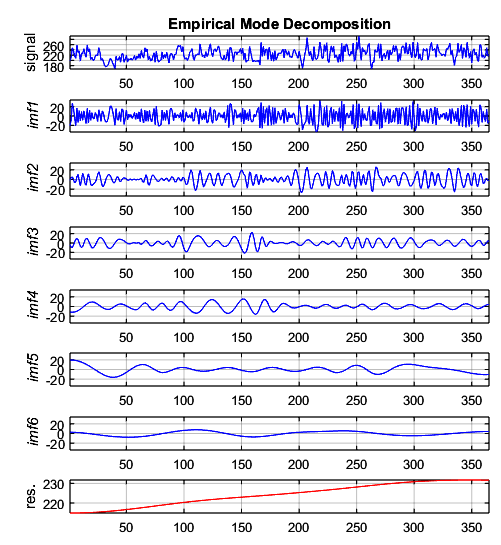


Fig. 3 Schematic diagram of the EMD components

*Step 2*: *Normalization*

Normalize sequence $\mathbf{z}_{k}=\{z_{k1},z_{k2},\cdots,z_{kT}\}_{k=1}^{N_{1}\text{+1}}$ as follows:

$\bar{z}_{ki}=\frac{z_{ki}-z_{k\min}}{z_{k\max}-z_{k\min}},i=1,2,\cdots,T,k=1,\cdots,N_{1}\text{+1}$,

and obtain the sequence $\bar{\mathbf{z}}_{k}=\{\bar{z}_{k1},\bar{z}_{k2},\cdots,\bar{z}_{kT}\}_{k=1}^{N_{1}\text{+}1}$.

*Step 3*: *Data phase space reconstruction*

To sufficiently extract the useful information from time series $\bar{\mathbf{z}}_{k}=\{\bar{z}_{k1},\bar{z}_{k2},\cdots,\bar{z}_{kT}\}_{k=1}^{N_{1}\text{+}1}$, the commonly used method is the phase space reconstruction (PSR) method in delay coordinates proposed by Packard et al.**^[20]^**. Theoretically speaking, a time series can sufficiently reconstruct an original dynamic system according to Takens**^[21]^**. From this procedure, time series $\bar{\mathbf{z}}_{k}=\{\bar{z}_{k1},\bar{z}_{k2},\cdots,\bar{z}_{kT}\}_{k=1}^{N_{1}\text{+}1}$ can be reconstructed in a multidimensional phase space as follows:

$\begin{matrix} \mathbf{x}_{ki}=\{\bar{z}_{ki},\bar{z}_{k(i+\tau)},\cdots,\bar{z}_{k(i+(m-1)\tau)}\}, \\ y_{ki}=\bar{z}_{k(i+m\tau)},i=1,\cdots,T-m\tau;k=1,\cdots,N_{1} \end{matrix}$(9)

where $\tau$ is the delay parameter and $m$ is the embedding dimension. It is very important to select a suitable pair of embedding dimensions $m$ and time delays $\tau$ when performing PSR**^[22-24]^**. Currently, there is no exact way to determine $\tau$ and $m$. It is advised that a larger value for $\tau$ than necessary should be selected to prevent system information from being ignored **^[25]^**. In the following, embedded dimension $m$ is set equal to 4 according Brock et al**^[26]^**, which indicates that the appropriate values for embedded dimension *m* should be between 2 and 5; the time delay is assumed to be day to day; i.e., $\tau=1$.

*Step 3*: *Data prediction with the IQPSO-LSSVM and representation*

The data pair $\{(\mathbf{x}_{ki},y_{ki})\}_{i=1}^{T^{*}}$ obtained in Eq. (9) is used to train the IQPSO-LSSVM and to obtain an optimal parameter pair ; then, the trained LSSVM is used to make a prediction

.

The final step is to carry out the reverse normalization on $\bar{\mathbf{z}}_{k}=\{\bar{z}_{k1},\bar{z}_{k2},\cdots,\bar{z}_{kT^{*}},\bar{y}_{k(T^{*}+1)},\cdots,\bar{y}_{k(T-m\tau)}\}_{k=1}^{N_{1}\text{+}1}$, which yields sequence $\mathbf{z}'_{k}=\{z'_{k1},z'_{k2},\cdots,z'_{kN}\}_{k=1}^{N_{1}\text{+}1}$ and obtains the forecast result $z_{j}^{p}=\sum_{k=1}^{N_{1}\text{+1}} z'_{kj},j=T^{*}+m\tau+1,\cdots,T$.

*Analysis of prediction results*

To evaluate the prediction accuracy, the dataset is partitioned into a training dataset (90%) and a validation dataset (10%). The training dataset can be applied to establish the prediction model, and the validation dataset can be applied to validate the effectiveness of the model.

First, the grey model (GM) and least squares support vector machine model (LSSVM) are used for the original dataset, and the prediction results are shown in Fig. 4, where the embedded dimension is set to $m=4$, and the time delay is day to day; i.e., $\tau=1$. The key parameters of the LSSVM are selected as $\gamma_{0}=30,\rho=0.1,\sigma=0.5$. It can be seen that the performance of the LSSVM is slightly better than that of the GM, but it is still not suitable for real applications.

To improve the prediction accuracy, first, EMD is applied to decompose the original vessel collision conflict sequence, and both the IMF components and residue are applied to establish the corresponding LSSVM models. The prediction of the LSSVM models with the same parameters for each IMF component are shown in Fig. 5, and the final prediction of the LSSVM for the original vessel collision conflict numbers is shown in Fig. 6. Notably, the performance of the EMD-LSSVM is better than that of the LSSVM. Therefore, from this point alone, EMD can significantly improve the prediction accuracy of the LSSVM. Of course, the prediction accuracy depends on the choice of the values of embedded dimension $m$, time delay $\tau$, and key parameters $\gamma_{0},\rho,\sigma$ of the LSSVM.


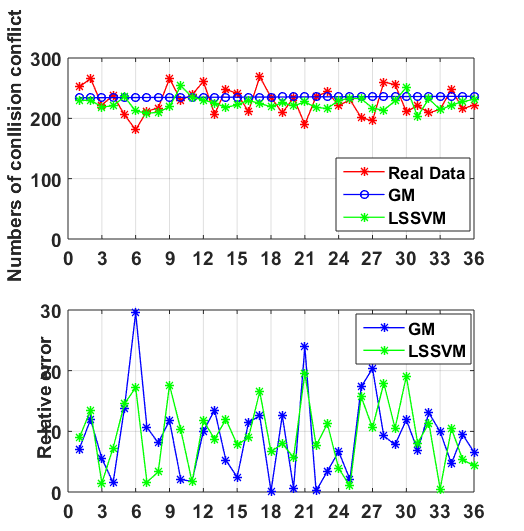


Fig. 4 The prediction results of the GM and LSSVM for the theoretical dataset.


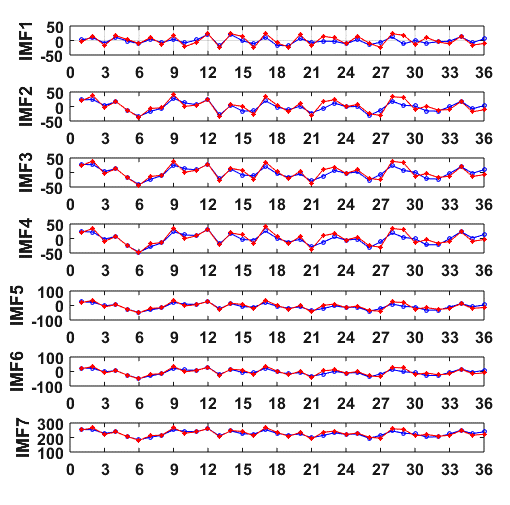


Fig. 5 The prediction of the LSSVM for each IMF component


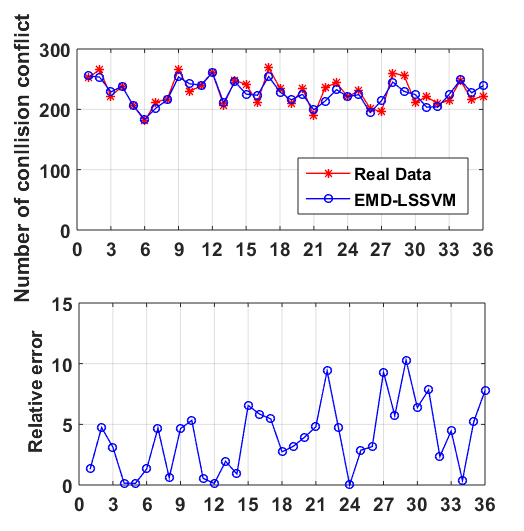


Fig. 6 The final prediction of the EMD-LSSVM for the original vessel collision conflict numbers

When the key parameters, $\gamma_{0},\rho,\sigma$, of the LSSVM are optimized by an improved quantum-behaved particle swarm, the final results and relative errors are shown in Fig. 7, from which it can be seen that the performance can be largely improved. Since there is no exact way to determine the best choice of $\tau$ and $m$, according to Brock et al**^[22]^**, different simulations are carried out to show the influence of embedded dimension *m* and the time delay on the error measures, as shown in Table 2 and Table 3.

Table 2 Influence of the embedded dimension on the error measures

| $e$ $m$ | $\text{e}_{\text{MAE}}$ | $\text{e}_{\text{MAPE}}$ | $\text{e}_{\text{MSE}}$ | $\text{e}_{\text{MSPE}}$ | $\text{e}_{\text{Max}}$ |
| --- | --- | --- | --- | --- | --- |
| 2 | 8.7437 | 3.8078 | 1.8189 | 28.4425 | 9.3275 |
| 3 | 6.3555 | 2.7544 | 1.2645 | 19.5252 | 6.2105 |
| 4 | 5.9771 | 2.5920 | 1.2455 | 19.2104 | 5.8652 |
| 5 | 7.3790 | 3.2108 | 1.5315 | 23.8872 | 7.6024 |

Table 3 Influence of the time delay on the error measures

| $e$ $\tau$ | $\text{e}_{\text{MAE}}$ | $\text{e}_{\text{MAPE}}$ | $\text{e}_{\text{MSE}}$ | $\text{e}_{\text{MSPE}}$ | $\text{e}_{\text{Max}}$ |
| --- | --- | --- | --- | --- | --- |
| 1 | 5.9771 | 2.5920 | 1.2455 | 19.2104 | 5.8652 |
| 2 | 6.3555 | 2.7544 | 1.2645 | 19.5252 | 6.2105 |
| 3 | 9.4927 | 4.1801 | 1.8114 | 28.8254 | 8.3482 |


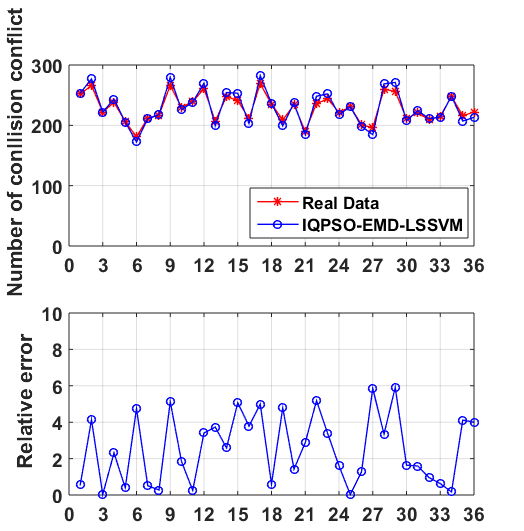


Fig. 7 The final prediction result of the IQPSO-EMD-LSSVM for the original vessel collision conflict numbers

V.  CONCLUSION

The Taiwan Strait is a large channel between northern and southern China and is an important maritime passage connecting the Korean Peninsula, Japan, Southeast Asian countries, Hong Kong and Macao. The ship traffic flow is large, the navigation risk is high, and the daily average number of vessel collision conflicts is approximately 220. The number of vessel collision conflicts is the main index for evaluating maritime traffic safety and measuring maritime management. It is of great significance for maritime administrative authorities to formulate strategies to reduce vessel collision accidents by predicting the occurrence of ship collision conflicts in the Taiwan Strait in a short period of time through historical collision conflicts. By considering the advantages of the LSSVM and QPSO and compensating for the limitation of the LSSVM in parameter solving, an integrated prediction model is proposed. The original vessel collision conflict time series are first decomposed into a collection of intrinsic mode functions (IMFs) and a residue with EMD. Second, both the IMF components and residue are applied to establish the corresponding LSSVM models, where the key parameters of the LSSVM are optimized by an improved quantum-behaved particle swarm optimization (IQPSO) algorithm. Then, each subseries is predicted using the corresponding LSSVM. Finally, the prediction values of the original vessel collision conflict datasets are calculated by the sum of the forecasting values of every subseries. The prediction results show that the EMD-LSSVM is an efficient algorithm and can be used in the forecasting of vessel accidents.

ACKNOWLEDGEMENT

This study is supported by the Natural Science Foundation of Fujian Province (Grant No. 2019J01326) and the Scientific Research Foundation of Jimei University, China.

CONFLICT OF INTERESTS

The authors declare that there are no conflicts of interest regarding the publication of the paper.

**DATA AVAILABILITY STATEMENT**

The data used to support the findings of this study are included within the article.

REFERENCES

1. Chai, T, Xiong, D.Q., Zhang, X.G, Weng, J.X, Modeling vessel collision frequency in open seas, Chinese Journal of Scientific Instrument 38(9), 2017, pp. 2335-2342.
2. Chai, T, Research on Risk Assessment of Ship Collision and Oil Spill Pollution based on Stochastic Methodology：The Case Study of Taiwan Strait, Dalian maritime university, 2018.
3. Lv, X.F, Yang, Y.D. Application of regression analysis method in the vessel traffic accident forecasting, Journal of Wuhan University of Technology 30(3), 2006, pp. 546-548.
4. Chen, Z.Y., Hu, S.P., Hao, Y.B., Prediction of marine traffic accidents based on fractal theory, Journal of Shanghai Maritime University 30(3), 2009, pp. 18-21.
5. Deng J.L., Introduction to grey system theory, The journal of Grey System 1, 1989, pp. 1-24.
6. Gan, H.X., Zhang B.G., Zheng, Y.Z., Peng, J.M., Application of the grey model theory to forecast maritime traffic accident, Ship & Ocean Engineering 37(6), 2008, pp. 99-102.
7. Chen, H.S., Wei, Q., Application of grey verhulst in water traffic accident forecasting, Navigation of China 36(2), 2013, pp. 67-69.
8. HuimingDuan, Xinping Xiao, and Lingling Pei, Forecasting the Short-Term Traffic Flow in the Intelligent Transportation System Based on an Inertia Nonhomogenous Discrete Gray Model, Complexity, Article ID 3515272, Volume 2017 (2017), 1-16.
9. Wei Meng, Daoli Yang, and Hui Huang, Prediction of China’s Sulfur Dioxide Emissions by Discrete Grey Model with Fractional Order Generation Operators, Complexity, Article ID 8610679, Volume 2018 (2018), 1-13.
10. HuimingDuan, GuangRong Lei, and Kailiang Shao, Forecasting Crude Oil Consumption in China Using a Grey Prediction Model with an Optimal Fractional-Order Accumulating Operator, Complexity, Article ID 3869619, Volume 2018 (2018), 1-12.
11. HuimingDuan and Xinping Xiao, A Multimode Dynamic Short-Term Traffic Flow Grey Prediction Model of High-Dimension Tensors, Complexity, Article ID 9162163, Volume 2019 (2019), 1-18.
12. Zhao, J.N., Wu, Z.L., Forecasting of maritime accidents by grey-Markov model, Journal of Dalian Maritime University 31(4), 2005, pp. 15-18.
13. Wang, Q. Wang, Z.M., Forecasting of maritime traffic accidents based on the improved SCGM(1,1)_c - Markov model, Navigation of China 36(4), 2013, pp. 119-124.
14. Suykens, J.A.K., Vandewalle, J., Least squares support vector machine classifiers, Neural Processing Letters 9(3), 1999, pp. 293-300.
15. Sun, J., Feng, B., Xu, W.B., Particle swam optimization with particles having quantum behavior, Evolutionary Computation 1(1), 2004, pp. 325-331.
16. N.E. Huang, Z. Shen, S.R. Long, M.C. Wu, H.H. Shih, Q.N. Zeng, et al, The empirical model decomposition and the Hilbert spectrum for nonlinear and non-stationary time series analysis, Proc. R. Soc. Lond. A – Math. Phys. Eng. Sci. 454 (1998) 903-995.
17. C.F. Chen, M.C. Lai, C.C. Yeh, Forecasting tourism demand based on empirical mode decomposition and neural network, Knowledge-Based Systems, 26 (2012), pp. 281-287.
18. Jujie Wang, Zhangli Ding, Forecaststing wind speed using empirical model decomposition and Elman neural network, Applied soft computing, 23, pp.45-459, 2014.
19. Sun J, Wu X J, Palade V, etc. Convergence Analysis and Improvements of Quantum- behaved Particle Swarm Optimization, Journal of Information Science 193, 2012, pp. 81-103.
20. Packard, N.H., Crutchfield, J.P., Farmer, J.D., Shaw, R.S., Geometry from a time series, Phys. Rev. Lett. 45, 1980, pp. 712-716.
21. Takens, F., Dynamical systems and turbulence, Berlin: Springer Verlag Press, 1981, pp. 366-381.
22. Kim, H.S., Eykholt, R., Salas, J.D., Nonlinear dynamics delay times and embedding windows, Phys D 127(1), 1999, pp. 48-60.
23. Ma, H.G., Han, G.Z, Selection of embedding dimension and delay time in phase space reconstruction, Front. Electr. Electron. Eng. China 1, 2006, pp. 111-114.
24. Kugiumtzis, D., State space reconstruction parameters in the analysis of chaotic time series e the role of the time window length, Phys D, 95(1), 1996, pp. 13-28.
25. Han, M., Prediction theory and method of chaotic time series, China Water & Power Press, 2007.
26. Brock, W.A., Hsieh, D.A., LeBaron, B., Nonlinear Dynamics, Chaos, and Instability: Statistical Theory and Economic Evidence, MIT Press, Cambridge, MA, 1991.
